# Analysis of Domain Architecture and Phylogenetics of Family 2 Glycoside Hydrolases (GH2)

**DOI:** 10.1371/journal.pone.0168035

**Published:** 2016-12-08

**Authors:** David Talens-Perales, Anna Górska, Daniel H. Huson, Julio Polaina, Julia Marín-Navarro

**Affiliations:** 1 Instituto de Agroquímica y Tecnología de Alimentos, CSIC, Paterna, Valencia, Spain; 2 Center for Bioinformatics, University of Tübingen, Tübingen, Germany; University of Colorado Anschutz Medical Campus, UNITED STATES

## Abstract

In this work we report a detailed analysis of the topology and phylogenetics of family 2 glycoside hydrolases (GH2). We distinguish five topologies or domain architectures based on the presence and distribution of protein domains defined in Pfam and Interpro databases. All of them share a central TIM barrel (catalytic module) with two β-sandwich domains (non-catalytic) at the N-terminal end, but differ in the occurrence and nature of additional non-catalytic modules at the C-terminal region. Phylogenetic analysis was based on the sequence of the Pfam Glyco_hydro_2_C catalytic module present in most GH2 proteins. Our results led us to propose a model in which evolutionary diversity of GH2 enzymes is driven by the addition of different non-catalytic domains at the C-terminal region. This model accounts for the divergence of β-galactosidases from β-glucuronidases, the diversification of β-galactosidases with different transglycosylation specificities, and the emergence of bicistronic β-galactosidases. This study also allows the identification of groups of functionally uncharacterized protein sequences with potential biotechnological interest.

## Introduction

βnGalactosidases are classified by the Enzyme Commission as EC3.2.1.23/108. They hydrolyze terminal, non-reducing D-galactosyl residues connected through e-glycoside linkages to polymers, oligosaccharides or secondary metabolites [1, 2]. These are important enzymes in the food and dairy industries because of their use in the manufacture of lactose free milk products. Enzymatic hydrolysis of lactose has also other applications, such as preventing cheese ripening or crystallization in refrigerated dairy foods, and treatment of cheese whey [2]. An emerging application is the use of β-galactosidases to synthesize prebiotic galactooligosaccharides (GOS) by means of their transglycosylating activity. This is allowed by the retaining mechanism of these enzymes, which proceeds through two steps. In the first one, lactose undergoes a nucleophilic attack and a covalent intermediate is formed between the galactosyl residue and the enzyme, releasing glucose. In the second step either a molecule of water or lactose may act as the acceptor of this galactosyl moiety resulting in final hydrolysis or in the synthesis of a trisaccharide, respectively. The structural conformation of the active site determines the transglycosylation/hydrolysis ratio of the enzyme and the chemical profile of synthesized GOS [3–6].

Enzymes with β-galactosidase activity are found in glycoside hydrolase families GH1, GH2, GH35 and GH42, according to the classification of Lombard et al. (2014) [7]. All these are grouped in clan GH-A, comprising mono and multi-domain proteins with a common (omm)8 TIM-barrel module. Only certain enzymes belonging to families GH1 and GH2 have lactose as natural substrate, whereas galactosidases belonging to families GH35 and GH42 act on different galactose-containing molecules, such as galactans [2, 8]

GH2 is a relevant enzyme family from both basic and applied points of view. The most conspicuous member of this family is the β-galactosidase encoded by the *lacZ* gene of *Escherichia coli*. This enzyme has been of paramount importance in the development of Genetics and Molecular Biology as reporter of gene expression [9]. It represents the first solved three-dimensional structure of the family and numerous site-directed mutagenesis studies have been carried out to elucidate the role of specific residues within its catalytic pocket [10]. Industrial applications of GH2 β-galactosidases include the aforementioned production of lactose-free milk products, for which lactase from *Kluyveromyces lactis* is used [1] and the production of chemically diverse GOS (reviewed in [5]). GH2 enzymes which synthesize GOS with different linkage types have been reported, such as the β-galactosidases from *K*. *lactis* (β-(1,6) GOS), *Bifidobacterium infantis* (β-(1,3) GOS), *Lactobacillus reuteri* (mixed β-(1,6) and β-(1,3) GOS) or *Bacillus circulans* (β-(1,4) GOS) [8, 11–13].

The GH2 family currently contains nearly seven thousand entries. Only a small fraction of the enzymes have been characterized functionally, most of which are β-galactosidases. Other activities, specifically β-glucuronidases, β-mannosidases, α-L-arabinofuranosidases, and exo-β-glucosaminidases have been identified [7]. The basic, simplest structure of GH2 enzymes, defined in Pfam [14] contains three modules: a TIM barrel domain that includes the catalytic residues, and two β-sandwich domains at the N-terminal end. Juers et al. (1999) [15] proposed an evolutionary relationship between GH2 β-galactosidases and other glycohydrolases using structural comparisons of the TIM barrel domain. This study suggested the existence of a primitive monodomain enzyme with a TIM barrel structure able to hydrolyze polysaccharides. Subsequent incorporation of the two β-sandwich domains at the N-terminal end rendered a modification of the catalytic site from a cleft-shaped conformation to a pocket-shaped one suited to hydrolyze small glycosides. This basic 3-domain composition of GH2 enzymes shows multiple variations. Most enzymes contain two additional, relatively well conserved β-sandwich domains at the C-terminal side of the catalytic module. We name enzymes with this composition "canonical β-galactosidases". In some cases functional β-galactosidases are bicistronic (encoded by two independent gene loci), composed of two subunits (LacL and LacM). LacL is homologous to the N-terminal β-sandwich domains and the TIM-barrel, whereas LacM is homologous to the C-terminal β-sandwich domain of canonical β-galactosidases. Some industrially relevant β-galactosidases, such as those from *Bacillus circulans* and *Bifidobacterium bifidum*, which have high transglycosylating efficiency [13, 16, 17], show a completely divergent modular arrangement at the C-terminal end. In the present work, we have carried out a bioinformatic analysis of the different architectures of GH2 β-galactosidases and the phylogenetic relationship among the catalytic domains. A detailed study of sequences related to the enzymes from *Bacillus circulans* and *Bifidobacterium bifidum* was carried out in order to identify new candidate enzymes able to produce GOS with high yield, based on sequence homology. The results of this analysis suggest that residues within the catalytic site potentially involved in transglycosylation are conserved in sequences with different domain architectures (DAs) but sharing a common BIG1-like non-catalytic module.

## Materials and Methods

### Analysis of domain architectures

GenBank accession numbers of protein sequences classified in the GH2 family were retrieved from the CAZy database [7] and mapped into the Uniprot database [18] using the tool “Retrieve/IDmapping” (http://www.uniprot.org/uploadlists/ (Accessed November 2015)). This operation yielded the UniProt code, GI number and Pfam domains of the different sequences, including the start and end coordinates of the domain limits (envelope) [14]. A binary vector of length 69 was assigned to each sequence. This vector indicates the presence (1) or absence (0) of a specific Pfam domain and its relative position in order from N- to C-terminus, including the existence of tandem repeats (Supplementary Material, S1 Annex). The amino acid sequences of the different proteins, in FASTA format, were retrieved from the NCBI database, using the Batch Entrez Tool (http://www.ncbi.nlm.nih.gov/sites/batchentrez (Accessed in November 2015). Finally, a matrix was generated that included the GI number, the binary vector, and the associated domain architecture (DA). We define DA as the linear composition of domains of a given sequence in N-terminal to C-terminal order. Only sequences that contained a single catalytic Glyco_hydro_2_C domain (GH2C), covering at least 70% of the corresponding Pfam consensus, were selected for further analysis. This set of DAs was refined to take into account C-terminal sequences corresponding to domains not identified by Pfam. We took into consideration the presence or absence of an extra C-terminal sequence, its length and similarity to establish a classification of the selected proteins. These protein sequences were also analyzed using the Interpro platform [19] in order to get information complementary to that provided by Pfam. The pipeline for DA analysis is summarized in the S1 Fig (supplementary information).

### Phylogenetic analysis

Sequence alignment was performed using ClustalO MSA [20] and CLC sequence viewer (QIAGEN). Trees were built using Maximum Likehood Parsimony algorithm with JTT matrix [21] and a bootstrap of 100 replicates using MEGA6 software [22]. Results were visualized on Dendroscope Software [23] and represented using FigTree software (http://tree.bio.ed.ac.uk/software/figtree/).

For the classification of proteins with unidentified C-terminal domains, these sequence stretches were extracted and aligned as described above. Subclusters (shown in S2 Annex and summarized in S2 and S3 Tables) were obtained from the resulting phylogenetic trees (not shown) with the exception of Cter1 sequences, included in DA type 5, which were classified in two subgroups, depending on the presence or absence of a Big1-like domain.

The final phylogenetic tree based on the sequence of the catalytic domain (GH2C) was constructed with a pool of 380 representative enzymes. A random selection of sequences of each specific domain architecture was carried out with DA types 4 and 5. In the case of DA types 1, 2 and 3 a previous subclustering was carried out (indicated in S2 Annex). Phylogenetic trees (not shown) including all the GH2C sequences of DA types 1 and 2 or, alternatively, DA type 3, were constructed as previously indicated to classify each group according to sequence homology. Sequences selected to construct the final tree included representatives of all subclusters.

### Docking analysis

Docking between β-D-(1,4)-galactosyl-lactose (β-D-galactopyranosyl-(1(1,4)-D-galactopyranosyl-(1(1,4)-D-glucopyranose) and *Bacillus circulans* β-galactosidase (PDB: 4YPJ) was carried out with the program Autodock4 (http://autodock.scripps.edu/references). The coordinates of β-D-(1,4)-galactosyl-lactose were obtained with GLYCAM (http://www.glycam.org). Using AutodockTools, hydrogens for ligand and receptor were added and charges were assigned with the Gasteiger method. All the hydroxyl bonds and glycosidic bonds were made rotatable, but not the sugar ring bonds. Autodock 4.0 was executed 100 times using Lamarckian algorithm (LGA), a population of 150, mutation rate of 0.02 and a crossover rate of 0.8. Simulations were performed using a maximum of 2500000 energy evaluations and a maximum of 27000 generations. Results of the docking were grouped using a cutoff value of 2 Å RMSD. The conformer with lowest binding energy was selected from a cluster which contained 42 out of 100 runs. Structural modelling and docking of *Thermotoga maritima* β-galactosidase with β-D-(1,3)-galactosyl-lactose has been previously described [4]. Pymol software (Delano Scientific LLC 2006) was used for structural analysis and visualization of modeled structures.

## Results

### GH2 domain architectures

**Fig 1:**
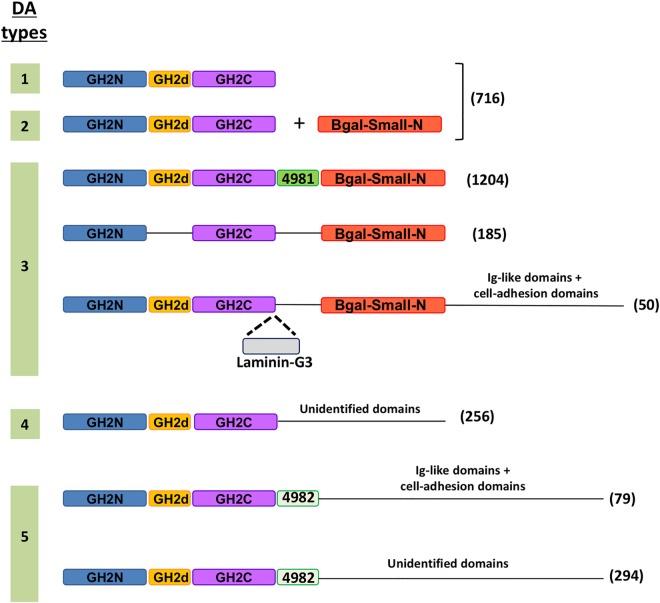
Domain architectures of GH2 family. Numbers in parentheses indicate the number of sequences representative of each DA type, included in the Pfam database.

The TIM barrel domain is the defining module of GH2 enzymes, since Glycoside Hydrolase classification is based on the sequence of their catalytic modules [7]. Our study of the GH2 family was carried out with proteins containing a canonical catalytic domain Glyco_hydro_2_C (GH2C), as defined by the Pfam database. Other GH2 sequences in which this Pfam domain was absent (e.g. mannosidases and arabinosidases) were excluded. Also, hybrid enzymes with additional catalytic modules (e.g. glycosyl transferases, lipases, deacetylases, kinases, hexoaminidase, etc.) were not considered. All the selected sequences contain two β-sandwich domains at the N-terminal side of GH2C, identified in Pfam as Glyco_hydro_2_N and Glyco_hydro_2 and abbreviated here as GH2N and GH2d, respectively (Fig 1). These domains correspond to the N-terminal modules described by Juers et al., (1999) [15] as characteristic of GH2 enzymes. In some cases, the GH2d domain is absent (DA type 3, Fig 1). However, these proteins contained instead a sequence of similar length to GH2d that likely represents an equivalent domain not defined in Pfam. Exceptionally, additional N-terminal domains related to carbohydrate recognition (Ricin B lectin domain) [24] or a cell surface adhesion signal (YSIRK signal) [25–27] were found, suggesting that these sequences correspond to surface anchored β-galactosidases.

The classification of DAs gave rise to five groups (DA types), summarized in Fig 1 and shown in detail in S2 Annex. One of the protein groups with higher number of representatives (DA type 1 with ca. 700 sequences) showed a DA constituted by the characteristic core of β-galactosidases (GH2N+GH2d+GH2C) without any additional domain. Most of these enzymes correspond to β-glucuronidases, according to current biochemical information and GenBank annotations, although these functional assignments need to be experimentally confirmed in most cases (Supplementary material, S1 Table and S2 Annex). A variation of this DA is represented by the large subunit (LacL) of bicistronic β-galactosidases (DA type 2). These enzymes have a bicistronic, heterodimeric constitution, formed by the interaction of LacL with a smaller subunit LacM (78 entries in Pfam, labelled Bgal_Small_N).

The most frequent topology (DA type 3) contains the Bgal_Small_N domain at the C-terminal end of the protein. These enzymes are mostly annotated as β-galactosidases in GenBank and only β-galactosidase activity has been reported for these enzymes, as registered in the CAZy database (Supplementary Material, S1 Table). Three different subtypes can be discerned. In most cases (1204 sequences) Bgal_Small_N is linked to the GH2C through a β-sandwich domain, of around 100 amino acids, identified in Pfam as DUF4981 (Domain of Unknown Function). In other cases (185 sequences) either DUF4981 or GH2d or both were absent, being replaced by sequences of similar length. In a small set of sequences (50 entries) probably corresponding to secreted enzymes, there are additional domains appended. For instance, in some proteins a Laminin G3 domain, related with cell adhesion, is inserted between the GH2C and the DUF4981 domains. Some sequences contain modular domains characteristic of extracellular proteins, such as NPCBM (novel putative carbohydrate binding module) or F5/F8 (discoidin domain), downstream the Bgal_Small_N domain [28, 29].

DA type 4 includes 256 proteins with a C-terminal (Ct) extension of 60–1053 residues without identified domains. We have classified these sequences according to the length and sequence similarity of the Ct extension (Supplementary material S2 Table). Around half of the GenBank annotations of these sequences do not specify any particular activity (Supplementary material S1 Table). Current biochemical characterization indicates that this is a heterogeneous group including β-galactosidase, β-glucuronidase, β-mannosidase and α-L-arabinofuranosidase activities.

**Fig 2:**
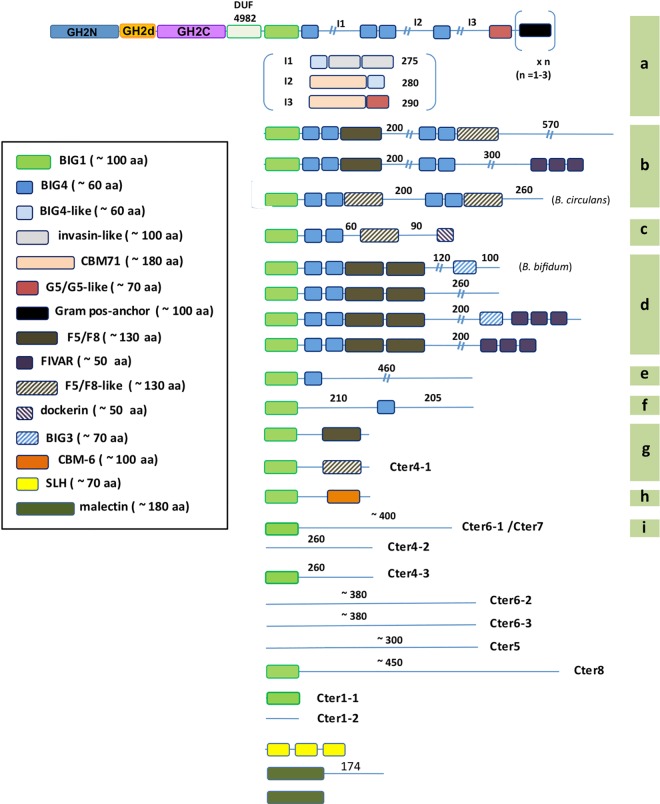
Domain architecture of DA type 5 sequences. Colored and stripped boxes correspond to domains identified by Pfam and Interpro databases, respectively. Modules with more than 40% sequence identity compared to BIG1 domains identified by Interpro, with a coverage higher than 60%, were tagged as BIG1. Letters (a-i) on the right edge of the figure group sequences with similar DAs. Domain assignment at the I1, I2, and I3 regions (subtype a) was based on the analysis carried out with the β-galactosidase from *S*. *pneumoniae* [30]. Numbers on top of non-identified regions indicate approximate number of residues.

DA type 5 (373 entries) includes proteins with the GH2C followed by the DUF4982 domain and additional C-terminal extensions (Fig 1). DUF4982 probably acts as a linker between GH2C and the C-terminal domains downstream, in a similar way that DUF4981 does in DA type 3 proteins. Most of these sequences are labelled as β-galactosidases in GenBank annotations and this is in fact the dominant activity among the characterized enzymes from this group (Supplementary material S1 Table). Most DA type 5 (294 sequences) contain C-terminal regions of 100 to 550 amino acids with unidentified domains. We have classified them according to the length and sequence similarity of the C-terminal region (Supplementary material, S3 Table). We considered that DA type 5 proteins deserved special attention because of the high variability of domain combinations at their C-terminal extension. Therefore, detailed analysis of this group was carried out that included domains described in Interpro [19], but not identified in Pfam. Results are shown in Fig 2. In a subset of these protein sequences, we detected different combinations of Ig-like domains that mostly occur in cell-wall anchored proteins and other modules involved in cell adhesion. Many of these sequences showed a BIG1-like domain downstream of DUF4982.

### Phylogenetic analysis of the GH2 catalytic domain

**Fig 3:**
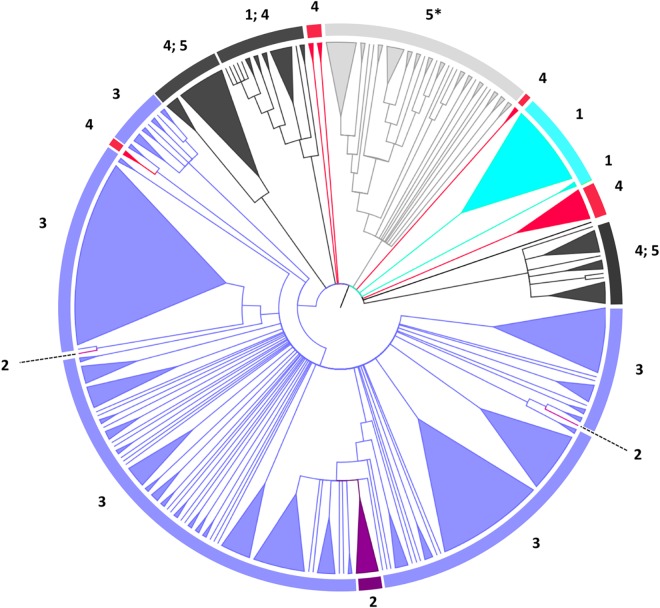
Phylogenetic analysis of the GH2C domain. The tree was calculated by Maximum Likelihood method based on the JTT matrix-based model [21] condensed at < 50% bootstrap support. The analysis involved a selection of 380 amino acid sequences for the different DAs. The tree was drawn using FigTree software (http://tree.bio.ed.ac.uk/software/figtree/). Numbers indicate the DA type. The asterisk marks the subtree further analysed in Fig 4. Sectors corresponding to a single DA type are colored in turquoise (DA type 1), purple (DA type 2), blue (DA type 3), red (DA type 4). Cluster 5* is colored in light grey. Sectors including mixed DA types are colored in dark grey.

The GH2C domain holds the nucleophile and acid/base catalysts and most of the residues that shape the active pocket. In order to analyze the impact of the different domain architectures on the evolution of the GH2C domain a phylogenetic tree was constructed using the GH2C modules of representative sequences for each DA. In the case of DA types 4 and 5, with multiple C-terminal domains, sequences were randomly selected for each specific domain architecture (Supplemetary Material, S2 Annex). Since DA types 1, 2 and 3, constitute the most numerous groups but show very little variation in the specific domain architecture, a previous subclustering of the GH2C domains was carried out in order to select a pool of sequences representative of all subclusters. It should be also considered that DA types 1 and 2 cannot be distinguished by the domain architecture of the gene carrying the GH2C module. Noteworthy, all sequences annotated in the GenBank as β-galactosidases large subunits (LS) were grouped in the same subcluster (numbered 4 in Supplementary material, S2 Annex). Sequences from subcluster 4 (both specifically annotated as LS or not) were selected and considered DA type 2 enzymes. In the final tree (Fig 3) all sequences corresponding to canonical β-galactosidases (DA type 3) cluster together. This cluster includes subtrees corresponding to bicistronic β-galactosidases classified as DA type 2. Interestingly, a subset of DA type 4 (labelled as Ct_8_3) also clusters within canonical galactosidases. A search in the Conserved Domain Database from NCBI [31], using the C-terminal region of these sequences as input, identified a sequence stretch of around 50–100 residues homologous to the central region of Bgal_Small_N domain. The remaining type 4 sequences form six different clusters. β-Glucuronidases (DA type 1) are dispersed in three different clusters. DA type 5 is divided in three clusters. Two of them contain members of DA type 4, whereas the other one, labeled 5* in Fig 3, shows a more complex structure that we describe below.

### Comparative analysis of enzymes with different transglycosylation properties

**Fig 4:**
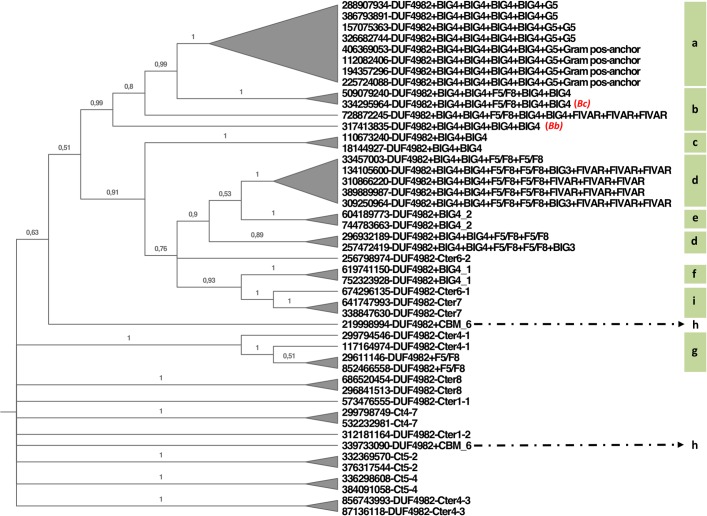
Phylogenetic subtree corresponding to the region marked 5* in Fig 3. Letters on the right edge of the figure group sequences with similar DAs, as indicated in Fig 2. Branch numbers indicate bootstrap values. Bc and Bf correspond to *Bacillus circulans* and *Bifidobacterium bifidum* β-galactosidases, respectively.

Enzymes with DA types 2 and 3 show high transglycosylating activity being able to synthesize β-(1,3) and β-(1,6) GOS [5, 32]. Commercial synthesis of β-(1,4) GOS is carried out with a β-galactosidase from *Bacillus circulans* [13, 33]. A related β-galactosidase from *Bifidobacterium bifidum* (BIF3) also shows high transglycosylating efficiency, although the chemical GOS profile has not been characterized [16]. Both enzymes belong to DA type 5. They carry a BIG1-like domain downstream the DUF4982 module (Fig 2) and their catalytic domains cluster under the same node (labelled 5* in Fig 3 and shown in more detail in Fig 4).

The structural determinants of transglycosylation of a DA type 3 enzyme, the β-galactosidase from *Thermotoga maritima* (TmLac) have been described elsewhere and, according to sequence homology, they may be extrapolated to other canonical and bicistronicβicistronic desc [4]. Moreover, the specific role of many of the residues involved in the activity of these enzymes can be inferred by multiple site-directed mutagenesis studies carried out with LacZ from *E*. *coli* [6, 10, 34–39]. On the contrary, information about structural-function relationships of enzymes from DA type 5 is scarce, with only two structures solved (BgaD-D from *Bacillus circulans* and BgaA from *Streptococcus pneumoniae*) [30, 40] and no site-directed mutagenesis studies within the catalytic pocket.

**Fig 5:**
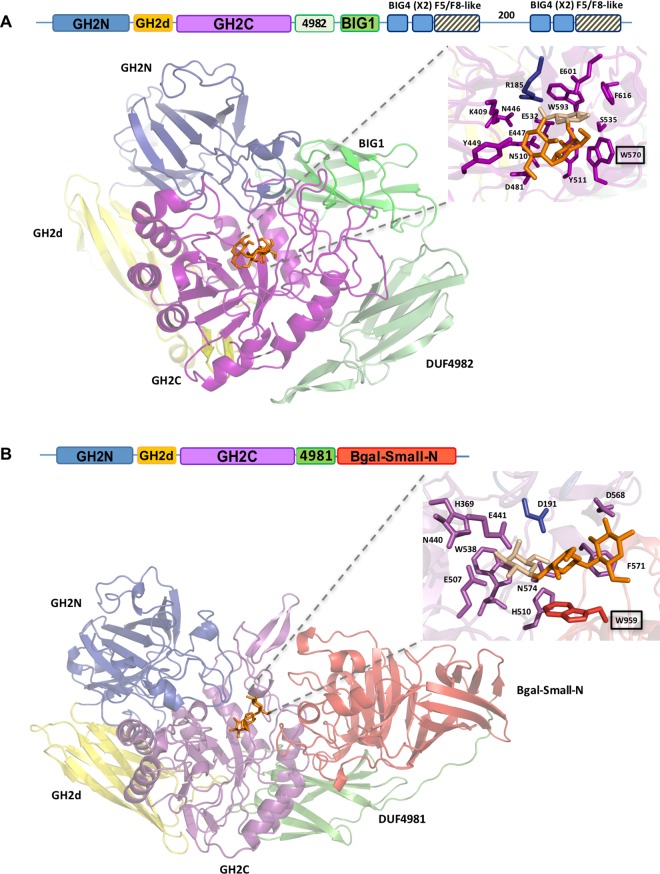
**Docking of a DA type 5 (A) and a DA type 3 (B) β-galactosidase with their main transglycosylation products.** The figure shows the domains that compose the architecture of the enzymes, represented in different colors. The residues potentially interacting with β-D-(1,4)-galactosyl-lactose in *Bacillus circulans* β-galactosidase (A) or with β-D-(1,3)-galactosyl-lactose in *Thermotoga maritima* β-galactosidase (B) are highlighted on the right side.

In order to relate the transglycosylating capability of industrially interesting enzymes with their phylogenetic position, a detailed study of DA type 5 was carried out. Amino acid residues within the catalytic pocket, including those potentially involved in transglycosylation, were identified by a docking study of the trisaccharide β-D-(1,4)-galactosyl-lactose into the *B*. *circulans* β-galactosidase structure (Fig 5A). This complex may be considered an analogue of the galactosyl covalent intermediate in the reaction mechanism, bound to the acceptor lactose. One of the residues interacting with this ligand is located within the GH2N domain, whereas the other 13 reside in the GH2C domain, including the catalytic triad formed by Glu 447 (acid base), Glu 532 (nucleophile) and Tyr 511 [40]. Most contacts would be established with the galactosyl moiety located deeper in the active site, which would be covalently linked to the enzyme in the reaction intermediate. Residues Trp 570, Asp 481, Tyr 449 and Lys 409 may be potentially involved in binding the acceptor lactose. A similar docking analysis with the previously studied TmLac in complex with its main transglycosylation product (β-D-(1,3)-galactosyl-lactose) is shown in Fig 5B [4]. According to this analysis, the residues building the catalytic pocket are completely different comparing both enzymes.

**Fig 6:**
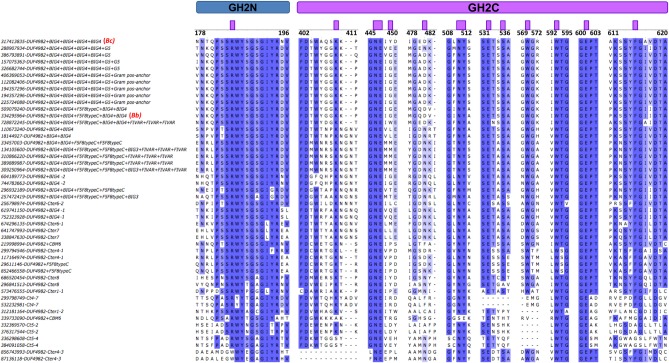
Sequence alignment around the putative catalytic site of proteins analyzed in Fig 4. Purple boxes indicate residues potentially involved in the active site, as predicted by docking analysis with β-D-(1,4)-galactosyl-lactose. Positions with more than 50% identity are colored in light blue and those with 100% identity are shown in dark blue color. Bc and Bf correspond to *Bacillus circulans* and *Bifidobacterium bifidum* β-galactosidases, respectively.

Fig 6 shows a sequence alignment of DA type 5* enzymes. Residues potentially involved in the catalytic pocket (marked by purple squares) are largely conserved among sequences containing the BIG1 domain. Exceptions are Tyr 449, which is poorly conserved, and Asp 481 that changes to Asn in many instances, including the β-galactosidase BIF3 from *B*. *bifidum*. Some DA type 5 β-galactosidases lacking the BIG1 domain, labelled Cter6-2, also show high identity at positions potentially involved in transglycosylation.

## Discussion

In this work we have analyzed the topology of GH2 enzymes and their phylogenetic relationships. Analysis of domain architectures (DAs) was carried out using Pfam and Interpro databases. Proteins have been classified in 5 DA types (Fig 1) according to C-terminal variable topologies. The three-dimensional structure of several enzymes of DA types 1 and 3 is known, such as the β-glucuronidases from *E*. *coli*, *C*. *perfringens* or *H*. *sapiens* [41–43], or the β-galactosidases from *Arthrobacter sp*, *E*. *coli* or *K*. *lactis* [6, 9, 44, 45], as well as part of the structures of the β-galactosidases from *B*. *circulans* [40] and *S*. *pneumoniae* [30] (DA type 5). These structures reveal that GH2C interacts with domains GH2N and with either Bgal_Small_N in DA type 3 proteins or BIG1 in DA type 5, through loops that shape the catalytic site. The close resemblance of Bgal_Small_N domains in monocistronic (DA type 3) and bicistronic (DA type 2) proteins indicates that these two types evolved from a common ancestor, either by gen fusion of LacL/LacM modules or by gene disruption from the canonical type. Phylogenetic analysis of GH2C domains (Fig 3) shows that bicistronic proteins emerge from branches of DA type 3, suggesting that they evolved from monocistronic sequences by gene disruption. The fact that bicistronic enzymes are dispersed in three different nodes may indicate that this was not a single evolutionary event. This gene disruption allowed the loss of the linker domain (primitive DUF4981).

Results shown in Fig 3 suggest that the C-terminal domains directed the evolution of GH2 β-galactosidases. The signature of DA type 5* proteins, whose node is represented in Fig 4 is the presence of a BIG1 domain whereas the signature of the canonical DA type 3 β-galactosidases is the Bgal_Small_N domain. Indeed, the DA type 3 cluster includes not only canonical and bicistronic (type 2) galactosidases but also a subset from type 4 carrying a C-terminal domain of around 400 amino acids (Ct_8_3), which is also related to Bgal_Small_N. This is in agreement with a close structural relationship between these C-terminal domains and the active site within the GH2C domain, that has been maintained through evolution. In contrast, β-glucuronidases (DA type 1) lacking C-terminal domains are dispersed in different nodes.

**Fig 7:**
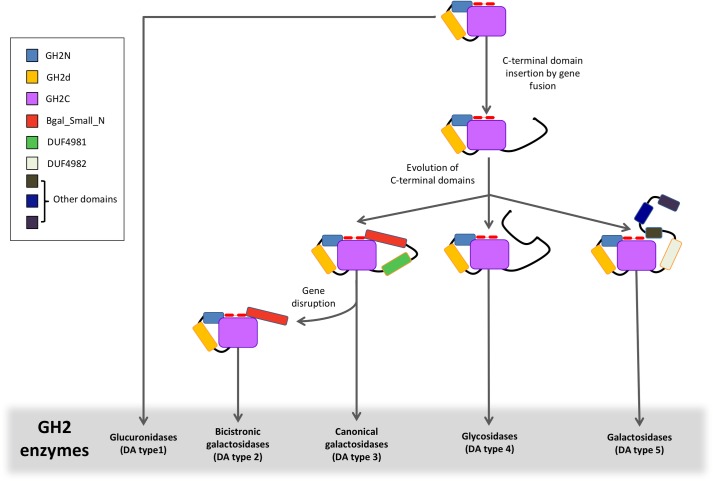
Proposed evolutionary model for GH2 enzymes.

We propose a model for the evolution of the different β-galactosidases DAs in family GH2, based on the results of phylogenetic analysis, which is presented in Fig 7. Primitive GH2 enzymes probably had the architecture GH2N+GH2d+GH2C (DA type 1) from which current glucuronidases derive. Subsequently, one or more C-terminal domains were added by gene fusion rendering types 3, 4 and 5. None of these was necessarily a single evolutionary event. A group of type 3 β-galactosidases gave rise to bicistronic β-galactosidases (type 2) by gene disruption. Likely, the addition of some of these C-terminal domains such as BIG1 or Bgal_Small_N gave shape to the active site and determined the specialization of GH2 enzymes in the hydrolysis of different galactosides. Other C-terminal domains seem to confer cell adhesion properties. For example, a module (CBM71) of the β-galactosidase from *Streptococcus pneumoniae* has been shown to be involved in host-pathogen recognition [46].

This study provides new insights that will help to screen and select novel β-galactosidases suited for specific GOS synthesis. Whereas β-(1,3) and β-(1,6) GOS are the main products of widely characterized β-galactosidases (classified in types 2 and 3), β-(1,4) GOS production has been described for the *B*. *circulans* β-galactosidase (type 5) [5]. This suggests that the divergent domain architecture of β-galactosidases may be related to the different product specificities of the transglycosylation reaction. Comparative docking analysis with a DA type 3 enzyme (TmLac) and a DA type 5 enzyme (BgaD-D) may add some hints about the structural basis for this different product specificity. Previous studies with TmLac showed that the main transglycosylation product of this enzyme is β-D-(1,3)-galactosyl-lactose, with a minor production of β-D-(1,6)-galactosyl-lactose [47]. Site-directed mutagenesis and structural modelling of TmLac suggested that Trp 959 (Fig 5B) functions as the aromatic binding platform for the acceptor lactose in the synthesis of the β-(1,3) GOS [4]. Although this Trp is not absolutely conserved within DA type 3 enzymes, it shows a high prevalence, and it is also found at an equivalent position in the LacM subunit of DA type 2 β-galactosidases [4]. Trp 959 in TmLac is located in a parallel plane to that formed by the galactosyl unit that would be transferred to lactose (Fig 5B). According to this, in TmLac (and other DA types 2 and type 3) this galactosyl moiety would be preferentially connected to equatorial hydroxyl groups from lactose, explaining the preference of these enzymes to synthesize β(1,3) linkages. The main transglycosylation product of the DA type 2 β-galactosidase from *K*. *lactis* is β-D-(1,6)-galactosyl-lactose [8]. This enzyme has a catalytic site very similar to that of TmLac except for the presence of a cysteine residue at the equivalent position of W959 (S3 Fig). In the case of BgaD-D, docking analysis shows a completely different structural arrangement for the catalytic site (Fig 5A). Trp 570 and Tyr 449 would be the candidate aromatic platforms for binding the acceptor lactose. However, sequence alignment of DA type 5 enzymes shows that Tyr 449 is located in a highly variable position (Fig 6), and it is not found in the β-galactosidase BIF3 from *B*. *bifidum* which also shows high transglycosylation efficiency [16]. On the contrary, Trp 570 is highly conserved in this set of sequences and may be the main anchoring site for the acceptor lactose. Interestingly, Trp 570 in BgaD-D is in a barely perpendicular plane to that of the galactosyl unit which is transferred to lactose (Fig 5A). Therefore, in BgaD-D, the galactosyl moiety would be mainly connected to the axial hydroxyl from lactose, resulting in a β(1,4) linkage. Sequence alignment (Fig 6) suggests that other DA type 5 enzymes containing a BIG1 domain, with a Trp at an equivalent position, may be good candidates for β-(1,4) GOS production, despite high divergence in C-terminal DA. Our analysis also shows the existence of a heterogeneous, poorly characterized group of proteins (DA type 4) with divergent GH2C sequences (Fig 3) and C-terminal ends (Supplementary Material, S2 Table, S2 Annex). Some of these sequences cluster together with DA types 1, 3 or 5 (Fig 3), which may reflect functional similarities. Other DA type 4 sequences are totally unrelated with any other group and may be considered good candidates in screening studies searching for enzymes with novel properties.

## Supporting Information

S1 Table Summary of Genbank annotations and biochemical characterization, as recorded in the CAZy database, of enzyme activity for each DA type. (DOCX)

S2 Table Cluster and subcluster classification of DA type 4 proteins. (DOCX)

S3 Table Cluster and subcluster classification of DA type 5 proteins with unidentified C-terminal extensions. (DOCX)

S1 Fig Pipeline followed for the analysis of GH2 domain architectures and phylogenetic tree construction. (TIF)

S2 Fig Phylogenetic analysis of the GH2C domain. The tree was generated as described in the legend of Fig 3, but including a tag for each sequence. The tag corresponds to the GI number and a descriptive legend of the corresponding domain architecture. In most cases, where the GH2N-GH2d-GH2C tandem is conserved, only the composition of the C-terminal domains downstream GH2C is indicated. When this does not occur, the full DA is shown. Within DA type 1 and 2 enzymes the specific subcluster of the GH2C domain is shown after the GI number. Sequences annotated in the GenBank as β-galactosidase large subunits are labelled as LS.
(PDF)

S3 Fig Structural superposition of the β-galactosidases from *Thermotoga*. *maritima* (TmLac) and *Kluyveromyces lactis* (KlLac). The structural model of TmLac (orange) was aligned with one of the subunits of KlLac (green, PDB code 3OB8). Residues contributing to the catalytic pocket are highlighted.
(TIF)

S1 Annex Binary vectors assigned to each sequence to describe the different domain architectures. Sheet S1.1 specifies the Pfam domain assigned to each position in the binary vector. Each Pfam code is followed by an underscore symbol and a number to indicate the presence of tandem repeats when necessary. Sheet S1.2 indicates the binary vector that corresponds to each sequence, identified by its GI number.
(XLS)

S2 Annex Domain architectures of all GH2 sequences analyzed in this study. Sequences are classified according to DA type. Each sequence is identified by the GI number and Accession number from Genbank, EMBL or DDBJ databases. The specific domain architecture, organism of origin (source) and Genbank definition is shown. Biochemical characterization, as recorded in the CAZy database, is also indicated. Within DA types 1 and 2, and also in DA type 3, the subcluster assigned to each GH2C domain according to phylogenetic analysis is indicated. In the case of unidentified C-terminal domains found in DA types 4 and 5, the corresponding signature is shown. Sequences selected to build the final GH2C phylogenetic tree (Fig 3) are highlighted in green.
(XLSX)
