# Supplementary material for: Analysis of Domain Architecture and Phylogenetics of Family 2 Glycoside Hydrolases (GH2)
Source: PLoS One. 2016 Dec 8;11(12):e0168035. doi: 10.1371/journal.pone.0168035 (PMC5145203; doi:10.1371/journal.pone.0168035)
Supplement: S1 Table — (DOCX) [file pone.0168035.s001.docx]

Table S1. Summary of Genbank annotations and biochemical characterization, as recorded in the CAZy database, of enzyme activity for each DA type.

| DA type | Genbank annotations | Biochemical characterization |
| --- | --- | --- |
| DA1 | 480 β-glucuronidases  29 β-galactosidases  18 β-glucuronidases/ β-galactosidases  99 unspecified | 17 β-glucuronidases  1 β-galactosidase |
| DA2 | 72 β-galactosidases  4 β-glucuronidases/ β-galactosidases  14 unspecified | 9 β-galactosidases |
| DA3 | 1155 β-galactosidases/lacZ  53 β-glucuronidases  231 unspecified | 52 β-galactosidases |
| DA4 | 90 β-galactosidases  26 β-galactosidases/ β-glucuronidases  7 β-glucuronidases  4 mannosidases  129 unspecified | 4 β-galactosidases  2 β-glucuronidases  1 β-mannosidase  1 α-L-arabinofuranosidase |
| DA5 | 233 β-galactosidases  33 β-galactosidases/ β-glucuronidases  107 unspecified | 11 β-galactosidases  1 β-glucuronidase |
