# Supplementary material for: Analysis of Domain Architecture and Phylogenetics of Family 2 Glycoside Hydrolases (GH2)
Source: PLoS One. 2016 Dec 8;11(12):e0168035. doi: 10.1371/journal.pone.0168035 (PMC5145203; doi:10.1371/journal.pone.0168035)
Supplement: S2 Table — (DOCX) [file pone.0168035.s002.docx]

**Table S2. Cluster and subcluster classification of DA type 4 proteins.**

| Cter signature | Length | Number of sequences | Subcluster identification  [number of sequences for each subcluster] |
| --- | --- | --- | --- |
| Ct1 | 60-100 | 47 | Ct1-1 [22], Ct1-2 [3], Ct1-3 [4], Ct1-4 [4] |
| Ct2 | 100-150 | 30 | Ct2-1 [6], Ct2-2 [6], Ct2-3 [2], Ct2-4 [2] |
| Ct3 | 150 – 200 | 21 | Ct3-1 [6], Ct3-2 [5], Ct3-3 [5] |
| Ct4 | 200-250 | 55 | Ct4-1 [2], Ct4-2 [26], Ct4-3 [2], Ct4-4 [7], Ct4-5 [7], Ct4-6 [2], Ct4-7 [3] |
| Ct5 | 250-300 | 35 | Ct5-1 [3], Ct5-2 [3], Ct5-3 [14], Ct5-4 [3] |
| Ct6 | 300-350 | 15 | Ct6-1 [2], Ct6-2 [5], Ct6-3 [3] |
| Ct7 | 350-400 | 19 | Ct7-1 [7], Ct7-2 [2], Ct7-3 [3] |
| Ct8 | 400-450 | 21 | Ct8-1 [5], Ct8-2 [3], Ct8-3 [2], Ct8-4 [5] |
| Ct9 | 450-500 | 2 | - |
| Ct10 | 500-550 | 2 | - |
| Ct11 | 800-850 | 7 | - |
