# Supplementary material for: Analysis of Domain Architecture and Phylogenetics of Family 2 Glycoside Hydrolases (GH2)
Source: PLoS One. 2016 Dec 8;11(12):e0168035. doi: 10.1371/journal.pone.0168035 (PMC5145203; doi:10.1371/journal.pone.0168035)
Supplement: S3 Table — (DOCX) [file pone.0168035.s003.docx]

Table S3. Cluster and subcluster classification of DA type 5 proteins with unidentified C-terminal extensions.

| Cter signature | Length | Number of sequences | Subcluster identification [number of sequences for each subcluster] |
| --- | --- | --- | --- |
| Cter1 | 100-150 | 217 | Cter1-1 [106], Cter1-2 [111] |
| Cter2 | 150 – 200 | 2 | Cter2-1 [1], Cter2-2 [1] |
| Cter3 | 200-250 | 2 | Cter3-1 [1], Cter3-2 [1] |
| Cter4 | 250-300 | 11 | Cter4-1[4], Cter4-2 [3], Cter4-3 [4] |
| Cter5 | 300-350 | 4 | - |
| Cter6 | 350-400 | 47 | Cter6-1 [45], Cter6-2 [1], Cter6-3 [1] |
| Cter7 | 400-450 | 5 | - |
| Cter8 | 500-550 | 6 | - |
