# Supplementary figures and images for: Analysis of Domain Architecture and Phylogenetics of Family 2 Glycoside Hydrolases (GH2)
Source: PLoS One. 2016 Dec 8;11(12):e0168035. doi: 10.1371/journal.pone.0168035 (PMC5145203; doi:10.1371/journal.pone.0168035)

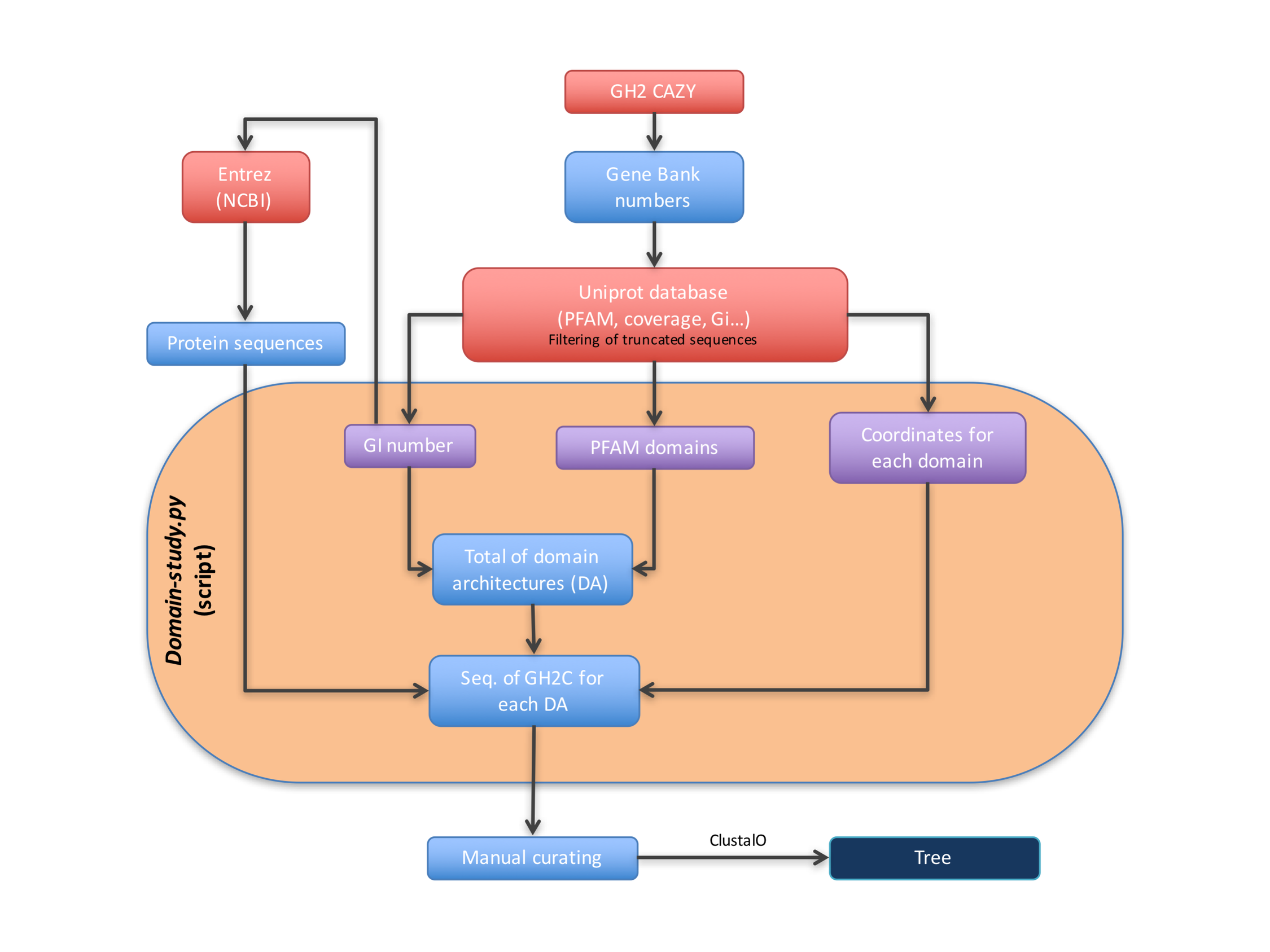

Supplement: S1 Fig — (TIF) [file pone.0168035.s004.tif]

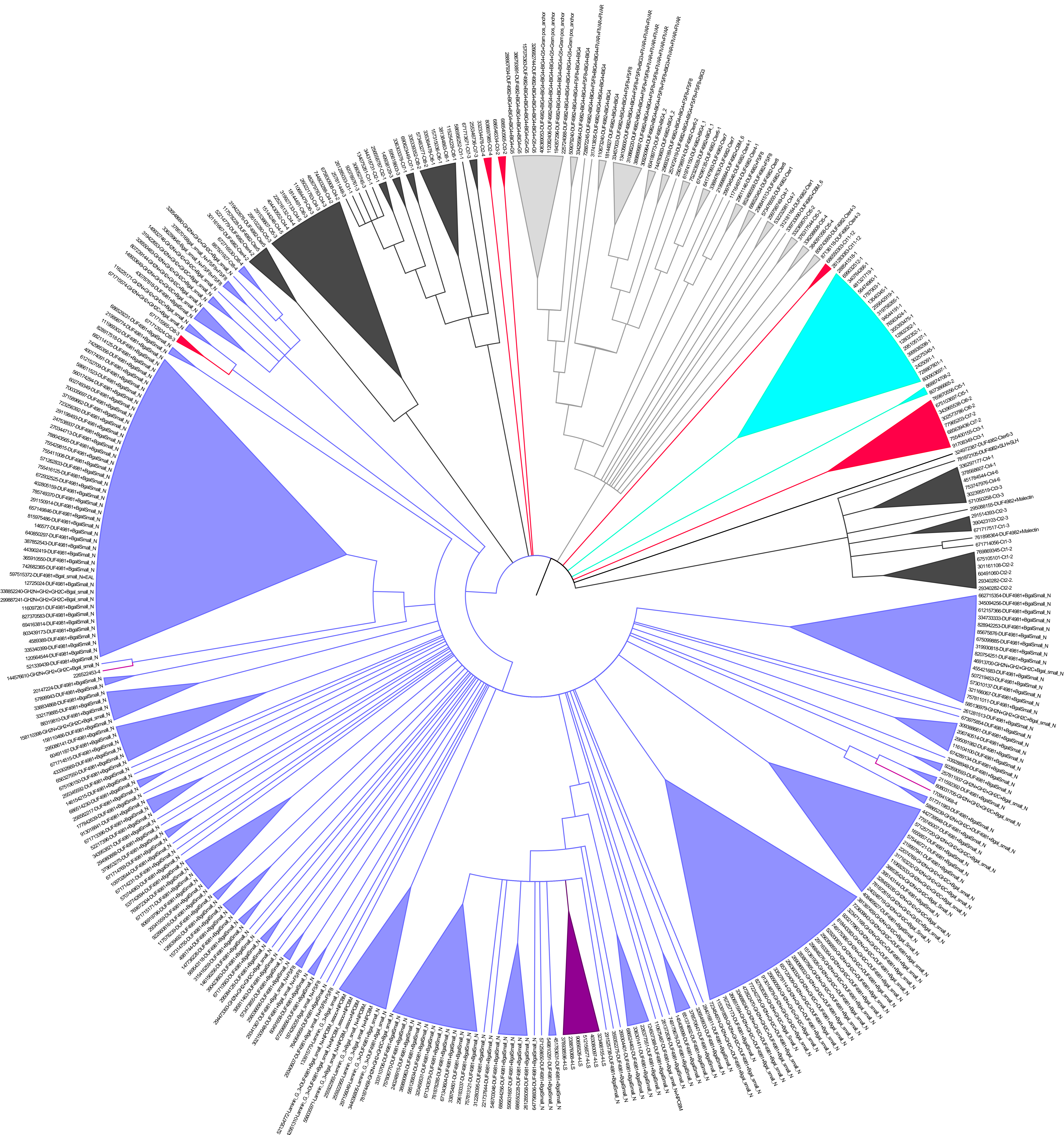

Supplement: S2 Fig — The tree was generated as described in the legend of Fig 3, but including a tag for each sequence. The tag corresponds to the GI number and a descriptive legend of the corresponding domain architecture. In most cases, where the GH2N-GH2d-GH2C tandem is conserved, only the composition of the C-terminal domains downstream GH2C is indicated. When this does not occur, the full DA is shown. Within DA type 1 and 2 enzymes the specific subcluster of the GH2C domain is shown after the GI number. Sequences annotated in the GenBank as β-galactosidase large subunits are labelled as LS. (PDF) [file pone.0168035.s005.pdf]

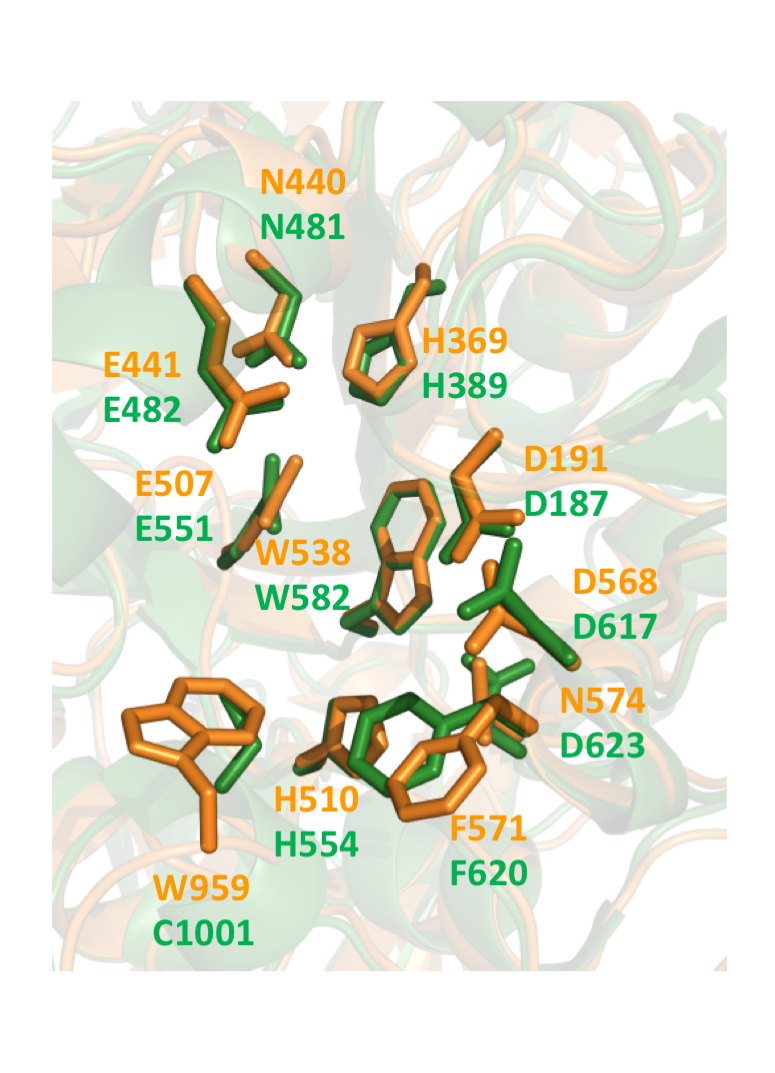

Supplement: S3 Fig — The structural model of TmLac (orange) was aligned with one of the subunits of KlLac (green, PDB code 3OB8). Residues contributing to the catalytic pocket are highlighted. (TIF) [file pone.0168035.s006.tif]
